# Genomes of *Conopholis americana* and *Epifagus virginiana*: two holoparasitic plants (Orobanchaceae)

**DOI:** 10.1093/g3journal/jkag169

**Published:** 2026-06-26

**Authors:** Marco Bürger, Adam C Schneider

**Affiliations:** Plant Biology Laboratory, Salk Institute for Biological Studies, La Jolla, CA 92037, United States; Howard Hughes Medical Institute, Salk Institute for Biological Studies, La Jolla, CA 92037, United States; Department of Biology, University of Wisconsin–La Crosse, La Crosse, WI 54601, United States

**Keywords:** Orobanchaceae, holoparasite, *Conopholis americana*, *Epifagus virginiana*, American cancer-Root, beechdrops, HiFi sequencing, genome assembly

## Abstract

*Conopholis americana* (American cancer-root) and *Epifagus virginiana* (beechdrops) are sister genera of holoparasitic plants (Orobanchaceae) native to eastern North America, parasitizing oaks and American beech, respectively. Both have served as models for plastid genome reduction, yet no nuclear genomes exist for either genus or any New World holoparasitic Orobanchaceae. Here we present the first nuclear genome assemblies for both species using PacBio HiFi sequencing. The *C. americana* assembly totals 1.82 Gb and *E. virginiana* totals 440 Mb, representing an approximately 4-fold difference in genome size between these sister genera. We observed a BUSCO completeness of 79% to 80% in both species, which is typical of holoparasites. While gene prediction identified 33,889 genes in *C. americana* and 21,031 in *E. virginiana*, repeat annotation revealed that LTR retrotransposons account for 78% of the genome size difference. These assemblies reveal contrasting mechanisms of genome evolution in sister holoparasitic genera and provide foundational resources for comparative genomics of parasitic plants.

## Introduction

The Orobanchaceae are the largest family of parasitic flowering plants, comprising approximately 90 genera and over 2,000 species that span the full spectrum of parasitic dependency, from facultative hemiparasites to obligate holoparasites entirely lacking photosynthesis. This diversity makes Orobanchaceae an important model system for studying the evolution of parasitism and its genomic consequences (Xu et al. 2022). Transcriptomic studies across the parasitism spectrum in Orobanchaceae have documented substantial changes in nuclear gene content associated with the transition to parasitism, including loss of photosynthetic gene expression in holoparasites, identification of core parasitism genes involved in haustorium development, and evidence for gene duplication and neofunctionalization as drivers of parasitic adaptation (Westwood et al. 2012; Yang et al. 2015). More recently, nuclear genome assemblies of Old World holoparasites have revealed that genome size varies considerably among closely related species while patterns of nuclear gene loss remain broadly consistent (Xu et al. 2022; Kim et al. 2024; Bürger et al. 2025).

**Fig. 1.**
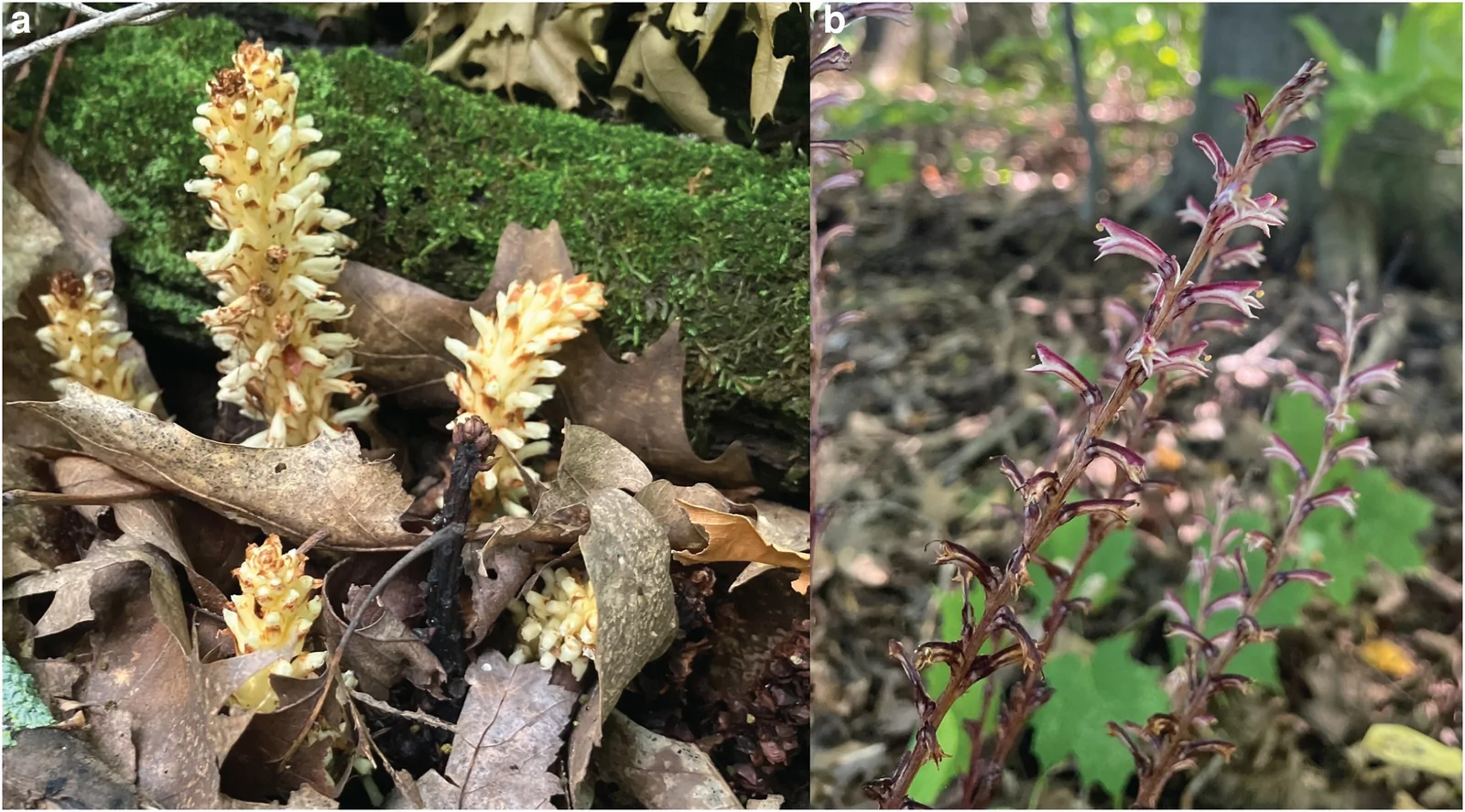
Species photographs. a) *Conopholis americana* population collected for this study (voucher *Schneider 1335*). b) *Epifagus virginiana* from iNaturalist observation 251028727.

Despite this progress, nuclear genomes are still absent for many important lineages, including all of those that have diversified in the New World. *Conopholis americana* (L.) Wallr. (American cancer-root) and *Epifagus virginiana* (L.) Barton (beechdrops) are holoparasitic plants native to eastern North America and represent sister genera within the family. *Conopholis americana* parasitizes red oaks (*Quercus* section *Lobatae*), in habitats with deep, moist soil, forming distinctive yellowish-brown flowering spikes emerging from host roots (Fig. 1; Percival 1931). *Epifagus virginiana* is an obligate parasite of American beech (*Fagus grandifolia*), with its distribution and postglacial migration closely tied to host density (Tsai and Manos 2010). Both species are nonphotosynthetic and obtain all water, nutrients, and carbon from their hosts via haustorial connections. Despite their close phylogenetic relationship, the two genera differ in chromosome numbers (2n = 40 in *C. americana*, 2n = 32 in *E. virginiana*), reflecting divergent karyotype evolution (Rodrigues et al. 2011).

These two species have been subjects of foundational molecular studies on organellar genome evolution in parasitic plants. *Conopholis americana* possesses one of the smallest plastid genomes reported from land plants at approximately 45 kb (Colwell 1994; Wicke et al. 2013), with loss or pseudogenization of photosynthesis-related genes but retention of ribosomal RNA genes (Wimpee et al. 1991, 1992). *Epifagus virginiana* also has a highly reduced plastid genome (∼70 kb), having lost all genes for photosynthesis and chlororespiration while retaining a minimal set of genes for plastid gene expression (Wolfe et al. 1992; Ems et al. 1995). These studies established both species as models for understanding plastid genome reduction under relaxed selective constraint. At the same time, like other holoparasitic Orobanchaceae, *C. americana* possesses an expanded mitogenome, exceeding 900 kbp (Feng and Wicke 2025). However, no nuclear genome assembly has been published for either species, leaving the evolutionary trajectory of most of the plant's genetic material an open question.

Here we report the first nuclear genome assemblies for *C. americana* and *E. virginiana*, generated using PacBio HiFi long-read sequencing. By comparing these sister genera, which share a holoparasitic lifestyle but differ, as we show here, in genome size by approximately 4-fold, we illustrate how genome size variation among holoparasites can be driven by transposable element dynamics as much as differential gene loss.

## Methods

### Species origin and sampling

Flower tissue was collected from a single individual of *Conopholis americana* and a single individual of *Epifagus virginiana*. Approximately 1 g of tissue was used for DNA extraction from each species. The specimen of *Conopholis* (voucher *Schneider 1335,* duplicates deposited at UWL and WIS herbaria, iNaturalist observation 218882486) was collected from Lower Narrows State Natural Area, Sauk County, WI, on 28 May 2024 under permit SNA 24-16. The specimen of *Epifagus* (*K.M. Wefferling 2098*, UWGB) was collected from Hagar Mountain State Natural Area, Oconto County, WI, on 21September 2024 under permit SNA 23-37B.

### DNA extraction and sequencing

High-molecular-weight genomic DNA was isolated using the Nanobind Plant Nuclei Kit (PacBio). Genomic DNA was quantified using a Qubit 3.0 fluorometer, and fragment size distribution was assessed using a Genomic ScreenTape on an Agilent TapeStation 4200. Samples meeting quality control guidelines (>50% of fragments >30 kb) were selected for library preparation. DNA was sheared using a Covaris g-TUBE centrifuged at 2,750 rpm in an Eppendorf 5430R to target 13 to 20 kb fragments. Sheared DNA underwent four full passes prior to cleanup with SMRTbell cleanup beads. SMRTbell libraries were prepared using the PacBio SMRTbell 3.0 kit following the manufacturer's protocol. Libraries were size-selected using 35% AMPure PB beads to remove fragments <5 kb. Final libraries were quantified using a Qubit 3.0 fluorometer and sized using an Agilent Femto Pulse with a Genomic DNA 165 kb kit. Libraries were loaded onto the PacBio Revio system at a targeted loading concentration of 200 to 300 pM per sample and sequenced for 30 hours per sample.

### Genome assembly

Raw HiFi reads were assembled into contigs using Hifiasm v0.24.0 (Cheng et al. 2021) with –primary and –write-ec flags for primary contig generation and error-corrected-reads output, respectively. Initially, a primary (collapsed) assembly was generated; however, we also generated haplotype-resolved assemblies to confirm that the primary assembly represents a single collapsed haplotype. Unless otherwise specified, downstream analyses used the primary assembly because a collapsed representation, which incorporates both haplotypes into a single consensus, is preferable for comparative genomics, repeat annotation, and completeness assessment where a single representative genome per species is required. Assembly statistics were calculated using assembly-stats v1.0.1. The script file for the assembly and annotation is provided in Supplementary File 1.

### Genome size estimation

K-mer counting was performed on error-corrected reads output by Hifiasm via the –write-ec flag, because error correction reduces spurious k-mers and improves genome size estimation accuracy (Xing et al. 2025), using Jellyfish v2.3.0 (Marcais and Kingsford 2011) with k = 21. K-mer frequency histograms were analyzed with GenomeScope v2.0 (Ranallo-Benavidez et al. 2020) to estimate genome size, heterozygosity, and repeat content. Ploidy was confirmed using Smudgeplot v0.2.5 (Ranallo-Benavidez et al. 2020), which analyzes the distribution of k-mer pair coverages.

### Decontamination

Assembled contigs were screened for contamination using NCBI FCS-GX v0.5.5 (Astashyn et al. 2024) (database build 2023-01-24) with taxonomic division set to plants. Contigs identified as foreign contamination were excluded from the final assemblies.

### Gene prediction and annotation

Protein-coding genes were predicted using Helixer v0.3.4 (Stiehler et al. 2021) with the land_plant model. Functional annotation was performed using funannotate v1.8.15 (Palmer and Stajich 2023) with the following databases: UniProt (v2023_05), InterProScan (v97.0), Pfam (v36.0), eggNOG-mapper, dbCAN (v12.0), MEROPS (v12.0), and Gene Ontology (v2023-11-15). Genome and annotation completeness were assessed using BUSCO v5.6.1 (Manni et al. 2021) with the eudicotyledons_odb10 database (2,326 BUSCOs) in protein mode.

### Repeat analysis

Transposable elements and repetitive sequences were identified and annotated using the Extensive de-novo TE Annotator (EDTA) v2.2.2 (Su et al. 2021). EDTA integrates multiple structure-based TE detection programs, including LTR_FINDER, LTR_HARVEST, LTR_retriever, TIR-Learner, HelitronScanner, and RepeatModeler. The pipeline was run with default parameters and used coding sequences (CDS) from the Helixer gene predictions to filter out gene fragments from the TE library. Whole-genome TE annotation was performed using RepeatMasker with the EDTA-generated species-specific TE library. TE divergence was calculated as the percent sequence divergence of each TE copy from the consensus sequence of its family.

### Strigolactone perception gene identification

Candidate D14 and KAI2 proteins were identified by BLASTp searches against the predicted proteomes of both species using *Arabidopsis thaliana* DWARF14 (AT3G03990) as query, with thresholds of E-value ≤0.0001, sequence identity ≥25%, and alignment length ≥220 amino acids.

## Results and Discussion

### Genome sequencing, decontamination, and assembly

For *Conopholis americana*, a single PacBio Revio run yielded 99.89 Gb of HiFi reads with a median read quality of Q36 (Table 1). The HiFi reads had a mean length of 15,124 bp and a median length of 14,519 bp. A total of 6,804,134 zero-mode waveguides (ZMWs) passed filters (37.91% of input), resulting in 6,604,283 HiFi reads. Of the total bases sequenced, 93.3% (93.22 Gb) had quality scores ≥Q30. For *Epifagus virginiana*, two PacBio Revio runs combined yielded 44.50 Gb of HiFi reads with a median read quality of Q37 (Table 1). The HiFi reads had a mean length of 15,733 bp and a median length of 14,880 bp. A total of 2,882,619 ZMWs passed filters (48.69% of input), resulting in 2,828,032 HiFi reads. Of the total bases sequenced, 95.5% (42.52 Gb) had quality scores ≥Q30.

**Table 1.** Sequencing statistics.

	*C. americana*	*E. virginiana*
Number of runs	1	2
Total HiFi yield (Gb)	99.89	44.50
Median read quality	Q36	Q37
Mean read length (bp)	15,124	15,733
Median read length (bp)	14,519	14,880
ZMWs passed filter	6,804,134	2,882,619
HiFi reads	6,604,283	2,828,032
Bases ≥Q30 (%)	93.3	95.5
Coverage (×)	54	104

Contamination screening identified markedly different profiles between the two species despite both samples coming from flower tissue. For *C. americana*, only 4 contigs (155,279 bp) were flagged, matching Pseudomonas phages (3 contigs) and *Pseudomonas aylmerensis* (1 contig). For *E. virginiana*, 312 contigs totaling 16,206,619 bp were identified as contamination. The majority matched nectar-associated yeasts (*Metschnikowia* spp.; 124 contigs), epiphytic ascomycete fungi (*Cladosporium colombiae*, *Melanodothis* sp., *Aureobasidium* spp.; 117 contigs), and plant-associated γ-proteobacteria (*Erwinia billingiae*, *Pantoea endophytica*, *Acinetobacter pollinis*; 69 contigs). Two contigs matched bacteriophages. The predominance of *Metschnikowia reukaufii* in *E. virginiana* is notable, as this yeast is commonly associated with floral nectar and is dispersed by pollinators; ants have been reported as the predominant pollinators of this species (Abbate and Campbell 2013). Similarly, *Cladosporium colombiae* and *Erwinia billingiae* are common plant epiphytes and endophytes. The contrasting contamination profiles between the 2 species likely reflect differences in environmental conditions at the time of collection or different pollination strategies, as *Conopholis americana* does not seem to be reliant on wind or insects for pollination (Baird and Riopel 1986). The detection and removal of these contaminants underscore the importance of thorough decontamination screening, particularly for field-collected plant samples.

The final decontaminated assembly of *C. americana* comprised 212 contigs totaling 1,823,809,973 bp (1.82 Gb), with an N50 of 93.31 Mb (L50 = 9), an N90 of 36.87 Mb (L90 = 21), and a largest contig of 119.12 Mb (Table 2). The final decontaminated assembly of *E. virginiana* comprised 201 contigs totaling 440,258,059 bp (440 Mb), with an N50 of 22.36 Mb (L50 = 9), an N90 of 17.12 Mb (L90 = 18), and a largest contig of 28.43 Mb (Table 2). The genome assemblies, gene models, and annotations of *Conopholis americana* and *Epifagus virginiana* are deposited in NCBI GenBank under BioProject PRJNA1424582. These contig-level assemblies are not scaffolded to chromosome scale; future Hi-C scaffolding would enable chromosome-level analyses.

**Table 2.** Assembly statistics for *C. americana* and *E. virginiana*.

	*C. americana*	*E. virginiana*
Total assembly size (bp)	1,823,809,973	440,258,059
Number of contigs	212	201
Largest contig (bp)	119,118,070	28,432,238
N50 (bp)	93,306,521	22,364,958
L50	9	9
N90 (bp)	36,867,400	17,120,844
L90	21	18
GC content (%)	35.29	35.31
Estimated haploid genome size (bp)	672,724,510	167,786,257
Heterozygosity (%)	9.75	11.9
Unique sequence (%)	43.3	63.8
Sequencing coverage (×)	54	104

### Genome size estimation

**Fig. 2.**
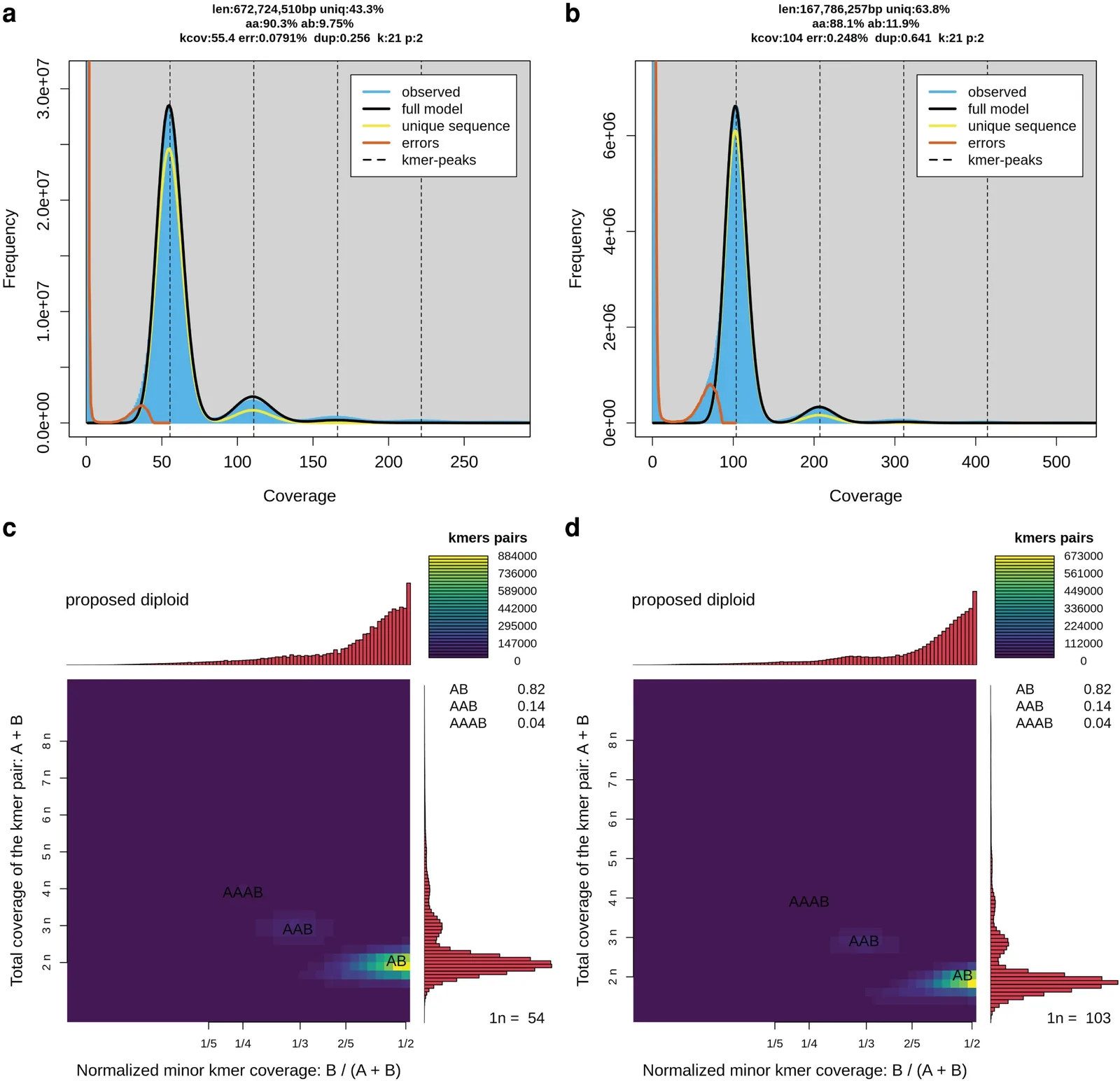
GenomeScope and smudgeplot profiles. a, b) GenomeScope k-mer profiles for *Conopholis americana* a) and *Epifagus virginiana* b), showing estimated genome size, heterozygosity, and repeat content (*k* = 21). c, d) Smudgeplot analysis confirming diploid genome structure for *C. americana* c) and *E. virginiana* d), with the majority of k-mer pairs falling in the AB (diploid) region.

GenomeScope analysis estimated the haploid genome size of *C. americana* at 673 Mb, with 9.75% heterozygosity and 43.3% unique sequence (Fig. 2a). However, the assembled size (1.82 Gb) is approximately 2.7-fold larger than the GenomeScope haploid estimate. Smudgeplot analysis confirmed the diploid genome structure, with 82% of k-mer pairs falling in the AB (diploid) category (Fig. 2c). The genome-wide heterozygosity estimate (9.75%) exceeds prior microsatellite-based estimates (average observed heterozygosity = 0.054) (Rodrigues et al. 2012), potentially reflecting the difference between genome-wide k-mer analysis and hypervariable microsatellite loci, or population-level variation.

GenomeScope analysis estimated the haploid genome size of *E. virginiana* at 168 Mb, with 11.9% heterozygosity and 63.8% unique sequence (Fig. 2b), but the assembled size (440 Mb) is approximately 2.6-fold larger than this estimate. Smudgeplot analysis similarly confirmed diploid genome structure (Fig. 2d). GC content was similar between species: 35.29% for *C. americana* and 35.31% for *E. virginiana*. The assembly sizes exceeding 2× the haploid estimates (2.7× and 2.6×, respectively) likely reflect limitations of k-mer-based genome size estimation in highly heterozygous genomes. GenomeScope's negative binomial model performs optimally at moderate heterozygosity levels (<5%) but can underestimate genome size in highly heterozygous genomes where the dominant heterozygous k-mer peak distorts model fitting. Both species substantially exceed this threshold (9.75% and 11.9%), possibly accounting for the discrepancy between k-mer estimates and assembly sizes. HiFi-based assemblies generally provide reliable size estimates and are likely closer to the true diploid genome sizes.

Haplotype-resolved assemblies confirmed that the GenomeScope underestimated haploid genome size, and the primary assemblies truly were a single collapsed haplotype (as opposed to partially uncollapsed diploid assemblies). For *E. virginiana*, the two haplotype assemblies were 456 Mb (hap1) and 432 Mb (hap2), closely matching the primary assembly size of 440 Mb. BUSCO genome-mode completeness was nearly identical across all three assemblies: primary (82.5% complete, 0.7% fragmented, 16.8% missing), hap1 (82.5% complete [78.0% single-copy, 4.5% duplicated], 0.7% fragmented, 16.8% missing), and hap2 (82.5% complete [77.6% single-copy, 4.9% duplicated], 0.7% fragmented, 16.8% missing). For *C. americana*, haplotype assemblies were 1,821 Mb (hap1) and 1,805 Mb (hap2), compared to the primary assembly of 1,824 Mb, with BUSCO genome-mode completeness of primary (81.7% complete, 0.6% fragmented, 17.7% missing), hap1 (81.4% complete [77.4% single-copy, 4.0% duplicated], 0.7% fragmented, 17.9% missing), and hap2 (81.6% complete [77.5% single-copy, 4.1% duplicated], 0.7% fragmented, 17.7% missing).

The haploid genome size of *E. virginiana* (∼440 Mb based on haplotype assembly sizes) places it among the smallest known Orobanchaceae genomes. Facultative hemiparasites such as *Castilleja* species have haploid genomes of 500 to 600 Mb (Bürger et al. 2024, 2026), obligate hemiparasites in the genus *Striga* range from ∼600 Mb to ∼2,460 Mb (Estep et al. 2012), holoparasitic *Orobanche* species range from ∼1.5 to 3.7 Gb (Xu et al. 2022; Bürger et al. 2025), and holoparasitic *Cistanche deserticola,* the closest relative to *Epifagus* and *Conopholis* with a fully sequenced genome, is 6.3 Gb (Zou et al. 2026). The small genome of *E. virginiana* is therefore unusual among Orobanchaceae and contrasts strikingly with its sister genus *C. americana* (haploid estimate 673 Mb) and close relative *Cistanche deserticola*, despite all 3 species sharing a holoparasitic lifestyle and variously reduced plastid genomes.

### Gene prediction and annotation

To determine whether the genome size difference reflects differential gene loss or other mechanisms, we analyzed gene content, BUSCO completeness, and repeat content for both species. Protein-coding genes were predicted using Helixer v0.3.4 with the land_plant model. 33,889 protein-coding genes were predicted in *C. americana* and 21,031 protein-coding genes were predicted in *E. virginiana* (Table 3). The higher gene count in *C. americana* is consistent with its larger genome size. For *C. americana*, predicted genes had an average length of 3,301 bp and an average protein length of 323 amino acids. Of the 33,889 genes, 28,381 (83.7%) were multiexon and 5,508 (16.3%) were single-exon transcripts. A total of 270,128 exons were predicted, with an average exon length of 207 bp. Functional annotation assigned GO terms to 18,796 genes (55.5%), InterProScan hits to 23,374 genes (69.0%), eggNOG annotations to 28,380 genes (83.7%), and Pfam domains to 18,063 genes (53.3%). For *E. virginiana*, predicted genes had an average length of 3,936 bp and an average protein length of 412 amino acids. Of the 21,031 genes, 17,770 (84.5%) were multiexon and 3,261 (15.5%) were single-exon transcripts. A total of 179,849 exons were predicted, with an average exon length of 247 bp. Functional annotation assigned GO terms to 15,110 genes (71.8%), InterProScan hits to 17,809 genes (84.7%), eggNOG annotations to 19,801 genes (94.2%), and Pfam domains to 15,298 genes (72.7%). Future refinement of the gene models with transcript evidence and homology-based approaches could further improve annotation quality.

**Table 3.** Gene annotation summary.

	*C. americana*	*E. virginiana*
Protein-coding genes	33,889	21,031
Average gene length (bp)	3,301	3,936
Average protein length (aa)	323	412
Multiexon transcripts	28,381	17,770
Single-exon transcripts	5,508	3,261
Total exons	270,128	179,849
Average exon length (bp)	207	247
Genes with GO terms	18,796 (55.5%)	15,110 (71.8%)
Genes with InterProScan hits	23,374 (69.0%)	17,809 (84.7%)
Genes with eggNOG annotations	28,380 (83.7%)	19,801 (94.2%)
Genes with Pfam domains	18,063 (53.3%)	15,298 (72.7%)

### Annotation completeness

**Fig. 3.**
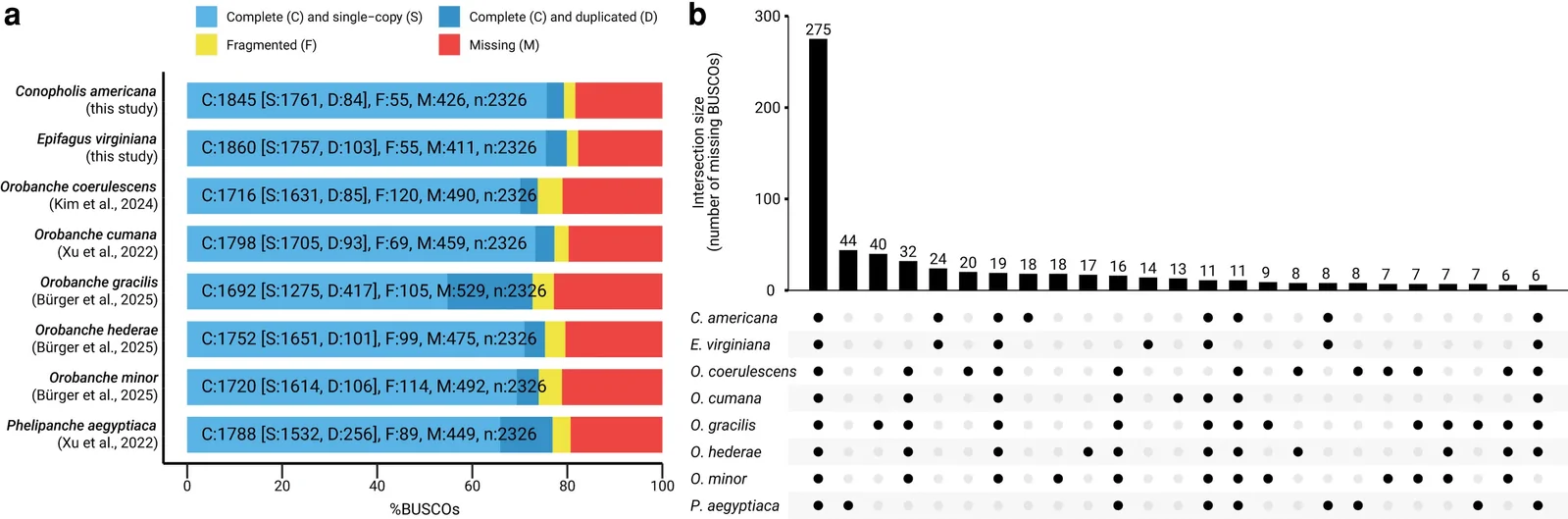
BUSCO completeness and shared gene loss across holoparasitic Orobanchaceae. a) BUSCO completeness assessment for *Conopholis americana* and *Epifagus virginiana* (this study) compared with published holoparasitic Orobanchaceae genomes using the eudicotyledons_odb10 database (*n* = 2,326). Species names are followed by literature citations. Complete BUSCOs are divided into single-copy (S) and duplicated (D) categories. b) UpSet plot showing the overlap of missing BUSCOs across all 8 species. Each vertical bar represents an intersection of species sharing the same missing BUSCOs, with the dot matrix below indicating which species participate. The largest intersection (275 BUSCOs) represents genes absent in all 8 species, indicating a conserved core of holoparasitic gene loss.

BUSCO analysis in protein mode indicated high completeness for both annotations (Fig. 3a). *C. americana* had 79.3% complete BUSCOs (75.7% single-copy, 3.6% duplicated), 2.4% fragmented, and 18.3% missing. *E. virginiana* had 79.9% complete BUSCOs (75.5% single-copy, 4.4% duplicated), 2.4% fragmented, and 17.7% missing. These completeness scores slightly exceed those of previously published holoparasitic Orobanchaceae genomes (Xu et al. 2022; Kim et al. 2024; Bürger et al. 2025), including *Orobanche* and *Phelipanche* species, which typically show 70% to 77% complete BUSCOs and 19% to 23% missing (Fig. 3a). An independent assessment using BUSCO in genome mode, which employs MetaEuk for protein-evidence-based gene finding directly on the assembly, yielded 81.7% and 82.5% complete BUSCOs for *C. americana* and *E. virginiana*, respectively, with near-identical missing percentages (17.7% and 16.8%). The concordance between these independent approaches confirms that the approximately 18% missing BUSCOs reflect genuine gene loss in holoparasites rather than annotation limitations.

To determine whether the missing BUSCOs represent shared or species-specific gene loss, we compared the missing BUSCO sets across eight holoparasitic Orobanchaceae species: *C. americana* and *E. virginiana*, five *Orobanche* species, and *Phelipanche aegyptiaca*. A core set of 275 BUSCOs was missing in all eight species, representing genes consistently lost across these samples (Fig. 3b). Between *C. americana* and *E. virginiana*, 370 of 426/411 missing BUSCOs were shared (87% to 90% overlap), indicating that these sister genera have lost largely the same gene set. This pattern is consistent with a previously observed holoparasitic gene loss of 363 shared missing BUSCOs between five Orobanche species (Bürger et al. 2025).

### Repetitive DNA

**Fig. 4.**
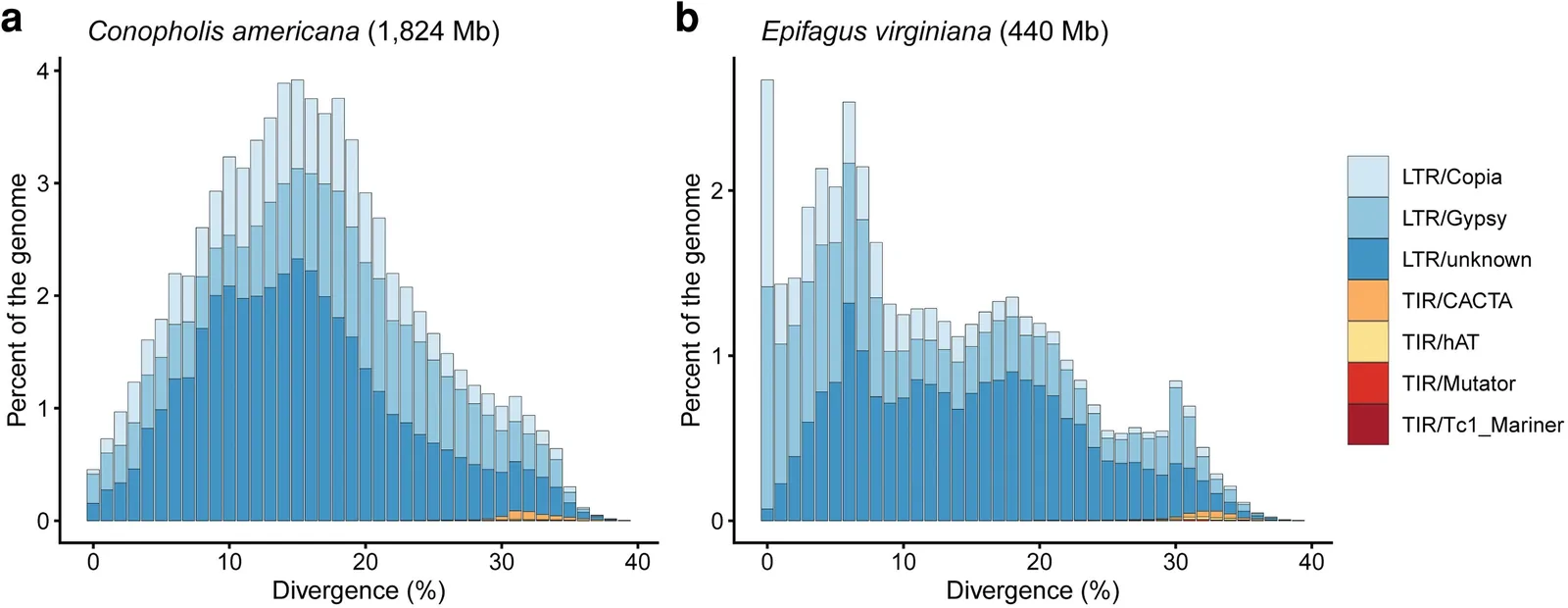
Repeat divergence landscapes. Distribution of transposable element copies by sequence divergence from consensus sequences for a) *Conopholis americana* and b) *Epifagus virginiana*. The x-axis shows percent divergence (proxy for relative age), and the y-axis shows the percent of the genome occupied by TE copies at each divergence level. LTR retrotransposons (Copia, Gypsy, unknown) dominate both genomes, but *C. americana* shows a peak at ∼15% divergence, while *E. virginiana* shows a peak at ∼5% divergence.

Repeat annotation revealed strikingly different repeat landscapes between the two sister genera (Table 4, Fig. 4). In *C. americana*, 69.07% of the genome (1,260 Mb) was identified as repetitive, compared to only 40.55% (179 Mb) in *E. virginiana*. LTR retrotransposons dominated both genomes but were far more abundant in *C. americana* (68.19% vs 39.29%), particularly the Gypsy (18.84% vs 13.66%) and Copia (13.69% vs 6.26%) superfamilies. A substantial fraction of LTR elements could not be classified to known superfamilies (35.66% in *C. americana*, 19.37% in *E. virginiana*), likely representing lineage-specific families. DNA transposons (TIR elements, Helitrons) and LINEs were minor components in both species, together comprising less than 1% of each genome. The approximately 4-fold difference in genome size between these sister genera (1,824 Mb vs 440 Mb) can be largely attributed to differential repeat content. Of the 1,384 Mb size difference, 1,082 Mb (78%) is accounted for by the difference in repeat content alone. The remaining 302 Mb of nonrepetitive sequence difference may reflect the higher gene count in *Conopholis americana* (33,889 vs 21,031 genes), longer intergenic regions, or other nonrepetitive sequences. Analysis of repeat divergence revealed distinct patterns of TE activity between species (Fig. 4). In *C. americana*, the divergence distribution peaked at approximately 15%, indicating that the majority of TE copies accumulated during an extended period of moderate TE activity in the past, with relatively little recent insertion. By contrast, *E. virginiana* showed a peak at lower divergence (0% to 5%), suggesting that the TE copies present are relatively recent, while older copies have been lost or were never accumulated.

**Table 4.** Repeat content summary.

Repeat class	*C. americana*			*E. virginiana*		
	Count	bp	%	Count	bp	%
**LTR retrotransposons**						
Copia	370,206	249,755,748	13.69	36,312	27,550,218	6.26
Gypsy	474,169	343,604,578	18.84	65,602	60,150,952	13.66
Unknown LTR	942,583	650,296,815	35.66	150,487	85,273,447	19.37
**DNA transposons (TIR)**						
CACTA	17,102	5,466,075	0.30	2,237	688,330	0.16
hAT	1,971	808,334	0.04	878	345,482	0.08
Mutator	1,015	360,784	0.02	387	133,888	0.03
Tc1/Mariner	28	11,703	<0.01	22	9,305	<0.01
PILE	229	66,367	<0.01	136	34,334	<0.01
POLE	389	221,703	0.01	168	94,471	0.02
**LINE (L1)**	7,375	5,486,574	0.30	3,679	2,545,127	0.58
**LINE (RTE)**	—	—	—	256	77,103	0.02
**Helitron**	3,167	1,080,557	0.06	820	276,301	0.06
**Repeat fragments**	15,218	2,485,947	0.14	8,288	1,353,373	0.31
**Total**	**1,833,452**	**1,259,645,185**	**69**.**07**	**269,272**	**178,532,331**	**40**.**55**

These findings indicate that while both the plastid and nuclear genomes of *E. virginiana* have undergone substantial reduction compared to related species, the mechanisms differ. The plastid genome reduction resulted from gene loss following relaxation of selection on photosynthesis genes. By contrast, the nuclear genome has retained largely the same set of conserved genes (a comparison of missing BUSCOs between the two species reveals 87% to 90% overlap (Fig. 3b)) but has undergone streamlining through reduced accumulation or active purging of LTR retrotransposons. The contrasting genome architectures of *E. virginiana* (smaller, streamlined), *Conopholis americana* (larger, repeat-rich), and *Cistanche deserticola* (largest and most repeat-rich; Zou et al. 2026), despite similar BUSCO completeness and shared holoparasitic ancestry, provide an opportunity for future comparative studies of genome size evolution in parasitic plants.

### Strigolactone perception

Parasitic plants in Orobanchaceae typically rely on strigolactone perception to detect host-derived germination signals. Mechanistically, variation in perception that impacts host specificity evolved through duplication and neofunctionalization of the ancestral KAI2 receptor, giving rise to the strigolactone-sensitive KAI2d clade, and *C. americana* was noted to possess only a single kai2 gene belonging to the slowly evolving kai2c clade (Conn et al. 2015). *Epifagus virginiana* similarly lacks KAI2d paralogs, and both species may have lost the need for strigolactone perception because their ectomycorrhizal host trees do not produce the strigolactones associated with arbuscular mycorrhizal plants (Merrilles et al. 2026). Our genome assemblies confirm these findings: both species contain a single copy of dwarf14 and a single copy of kai2c, and we were unable to detect any kai2d genes using *Arabidopsis* DWARF14 in both protein BLAST against the proteomes and in tBLASTn searches directly against the genome assemblies. The germination cues used by *C. americana* and *E. virginiana* in nature remain unknown. *Conopholis alpina* has been reported to germinate in response to the synthetic strigolactone GR24 and the smoke-derived butenolide, which shares structural similarity with the strigolactone D-ring (Daws et al. 2008). The germination response observed in *C. alpina* may reflect karrikin-type signaling through KAI2c rather than true strigolactone perception. Alternatively, *C. alpina* may have retained KAI2d, unlike its North American congener. Seeds of another close relative, *Kopsiopsis hookeri*, do not germinate in response to chemical cues at all, but rather the host root appears to mechanically penetrate the testa following a chance encounter (Olsen and Olsen 1981).

## Supplementary Material

jkag169_Supplementary_Data

## Data Availability

The genome assemblies, gene models, and annotations of *Conopholis americana* and *Epifagus virginiana* are deposited in NCBI GenBank under BioProject PRJNA1424582. The script file used for assembly and annotation is provided as Supplementary File 1. Supplemental material available at G3 online.
